# Attack and Defense Performance in Goalball: A Proposal for Throwing, Balance and Acoustic Reaction Evaluation

**DOI:** 10.3390/biology11081234

**Published:** 2022-08-18

**Authors:** A. Vanessa Bataller-Cervero, Pablo J. Bascuas, Juan Rabal-Pelay, Héctor Gutiérrez, Eduardo Piedrafita, César Berzosa

**Affiliations:** Facultad de Ciencias de la Salud, Universidad San Jorge, Autov. A-23 Zaragoza-Huesca, km 299, 50830 Villanueva de Gállego, Zaragoza, Spain

**Keywords:** goalball, balance, ball velocity, accuracy test, stabilometry, core stability, acoustic reaction time

## Abstract

**Simple Summary:**

Goalball is a dynamic sport for visually impaired people, where attack and defense situations alternate during the game. In attack situations, the throwing performance could be dependent on several variables such as the anthropometry, the core stability, or the balance of the athletes. In defense situations, the reaction time to an acoustic stimulus could be also considered as a performance factor. All these variables have been analyzed in this pilot study assessing the ball velocity and accuracy in throwing, analyzing the relationship with the previous parameters. New evaluation tests have been proposed in this research, suggesting a more specific evaluation test battery for this sport.

**Abstract:**

Goalball is a sport for visually impaired athletes, where the roles of attack and defense change continuously during the game. Performance evaluation should consider the variables that determine the throwing and the stop and clearance of the ball. The aim of this study is to evaluate the precision and velocity of the ball throwing in goalball, besides core stability and balance as variables that determine an optimal throwing. Moreover, a novel acoustic reaction time is applied to analyze the defense performance. Eight goalball players (33 ± 9 years old; 77.8 ± 22.7 kg; 174 ± 13 cm; 10 ± 5 years of experience) were recruited to assess ball velocity, with a radar gun, and throwing accuracy. Anthropometry, static balance, and core stability were assessed using a computerized pressure platform. Acoustic reaction time was measured with a photoelectric system. A significant positive correlation was found between throwing speed and the years of experience (Ƿ = 0.714, *p* = 0.047), height (Ƿ = 0.786, *p* = 0.021), dominant leg surface area of the stabilogram (Ƿ = 0.738, *p* = 0.037), and non-dominant leg center of pressure mean velocity (Ƿ = 0.714, *p* = 0.017). In the present pilot study, height and years of experience are correlated to throwing velocity. This is also the first test proposal to assess throwing precision and complex acoustic reaction in goalball players, which could be used to assess the level of performance in future studies.

## 1. Introduction

Goalball is a Paralympic sport played by blind or visually impaired people. It was designed as a part of the therapy for blinded war veterans by Hanz Lorenzen and Sepp Reindle in 1946. The game became a more competitive sport during the 1950s and 1960s, when players improved their attack and defense skills. Toronto 1976 was the debut of this sport in the Paralympic Games, and, in 1978, the first World Championships were held in Austria. At the moment, there are continental (Americas, Asia-Pacific, European, and African) Championships periodically, goalball being a high-performance sport [1]. It is a collective sport involving two three-player teams that play on a volleyball court. To avoid differences among players, they must wear blackened eye goggles that completely cover their eyes. It is played with an acoustic sensory ball with bells inside, and the aim of the game is to throw it to the opposing team’s goal line to score. Goalball players must develop a high level of acoustic and spatial awareness [2]. Each match has a 24 min total duration, which is divided in two halves of 12 min, although the match can end earlier if a team reaches an advantage of 10 goals [3]. In this game, the ball is thrown from a zone of 6 m, located at 12 m from the goal, with a width that is 9 m. Several shot types can be found in goalball, according to the player position at the moment of throwing. From the tactical point of view, the most effective shot types have been previously studied [4]. However, only tactical issues were analyzed in this research. In other studies, different shot techniques and their impact on ball velocity have been evaluated [5]. The precision and velocity of the ball are both determinants in a sport where goals are essential, so they must be evaluated to test the level of these athletes.

Different physical skills could determine the throwing performance. Upper and lower limb strength is a determinant in the game due to its relevance in throwing [6,7]. A significant relationship between seated medicine ball throw and throwing velocity has been found in previous studies in goalball players [8]. Core muscles, responsible for maintaining spine and pelvis stability, are also considered relevant in overhead throwing. They allow the transfer of force from the lower to the upper limbs [9]. In goalball players, core stability training has shown a positive effect on throwing velocity [10]. Other researchers have found that rotating throws allow for a higher ball velocity [5]. In this kind of movement, postural control plays a significant role, and core strength is essential for an optimum performance. Some authors consider core strength as synonym for core stability [11,12]. Core stability could be defined as the capacity of the muscles and joint structures coordinated by the motor control system, to maintain a position or trunk trajectory when it suffers from inner and outer forces. This ability has been related to lower limb injury prevention [11]. Core strength is important not only for injury prevention but also to improve other physical qualities. In other sports such as football, core training improves quickness and agility [13]. In addition, core strength could improve balance and preventing the risk of falling in elderly people [14].

Apart from core stability, there are other sensory systems that are very important to maintain postural control. They provide information about the body position, allowing to generate motion responses to maintain postural control [15]. In the process of balance control, vision plays a very important role. Visual input provides information to maintain posture. Visual disability is related to a dysfunction in movement, which affects the static balance [16]. Balance has been evaluated in visually impaired athletes, and the results show that sighted people with eyes closed have better results than blind athletes, while blind goalball players were better than sedentary blinds [17]. In visually impaired people, a period of core training improved their balance [18]. Therefore, regarding goalball, a higher core stability would allow a higher precision and throwing velocity, increasing the possibility of scoring goals. Hence, evaluation of core strength and balance could add value to the knowledge of the performance factors in goalball. Different tools could be found to analyze balance (accelerometers [19], pressure platforms, force platforms) or even measuring without technology (Flamingo test). Those that could offer quantitative data could be applied to the evaluations of this study. 

Several research studies have focused on the performance of athletes on different physical tests [2] and the relationship of certain physical skills with a velocity ball [8], but up to now no study has considered the performance parameters for both attack and defense in goalball players. Additionally, the velocity without precision has no sense in goalball, so both variables—velocity and precision—must be evaluated for throwing.

In goalball, the athletes change their role continuously from attackers to defenders. Performance variables in both roles are different. Meanwhile the velocity of throwing, precision, and balance are of outstanding value in attacks, while when the players are defending other performance factors must be considered. Defenders must avoid the ball passing the goal-line. The speed of the ball could be very high, so players must be vigilant and ready to jump. Due to the visual privation, goalball players must rely on their auditory system to detect the position and velocity of the ball. The speed of reaction to acoustic stimuli could be considered as a performance factor, so this should also be included in a battery test of performance evaluation in goalball. The closer the test is to reality, the more reliability it will have in the evaluation of the performance. Therefore, a test that includes real game movements should be considered.

Thus, the aim of this study is to evaluate the precision and the velocity of the ball throwing in goalball and analyze its relationship with several performance variables for attack and defense, being core stability, balance, acoustic reaction velocity, and anthropometry. This proposal of an evaluation test could be a very useful battery test for a complete evaluation of goalball athletes’ performance. Due to the small sample size, this study will be considered as a pilot study.

## 2. Materials and Methods

### 2.1. Study Design

This research is a cross-sectional study that could be considered as pilot study considering the small size sample. The descriptive study comprised two sessions of assessment, which were separated by one week. On the first day, informed consent form was signed after the objectives and procedures of the study were explained to the participants, with the collaboration of the researchers and ONCE collaborators. In addition, personal data were collected with a Personal Questionnaire Form (i.e., age, degree of disability, goalball training experience, injuries suffered), body composition was evaluated, and participants were familiarized with each test. During the second day, players were evaluated for acoustic reaction test, throwing accuracy, ball velocity, core stability and balance.

### 2.2. Participants

Eight goalball players (33 ± 9 years old; 77.8 ± 22.7 kg; 174 ± 13 cm; 10 ± 5 years of experience) participated in this study. Inclusion criteria were: (1) goalball players included in the Spanish Federation of Sports for Blinded People (Federación Española de Deportes para Ciegos); and (2) playing during the 2021–22 season in the highest category. The exclusion criteria were: (1) athletes that suffered neuromuscular or cardiovascular diseases within the three months before the study. All participants were recruited from a Spanish goalball team of the National Organization of Spanish blind people (Organización Nacional de Ciegos Españoles: ONCE). The athletes signed an informed consent, and the study was approved by the ethical committee of the institution (code 06/1/21-22). The subjects were classified into three groups according to the visual impairment degree: B1 (totally or almost totally blind) (*n* = 1), B2 (visual acuity below 2/60 and/or a visual field constricted to a diameter lower than 5 degrees) (*n* = 3), and B3 (partial sight, with visual acuity ranging from 2/60 to 6/60 and/or a visual field constricted to a diameter from 5 to 20 degrees) (*n* = 4) [20]. Their training period is based on 2 sessions/week, 150 min/session. The sessions always included a warm-up with jogging and specific exercise of throwing and defense. Strength exercises and game situations completed the sessions.

### 2.3. Body Composition

Body mass (kg) was measured with a SECA calibrated digital scale (model 799, Seca Corp, Hanover, MD, USA), with a precision of 0.1 kg and a range of 2–200 kg. Height (cm) was measured using a SECA stadiometer (model 206, Seca Corp, Hanover, MD, USA), with a precision of 1 mm and a range of 130–210 cm. Subsequently, body mass index (BMI) was calculated.

### 2.4. Throwing Accuracy and Ball Velocity

**Figure 1:**
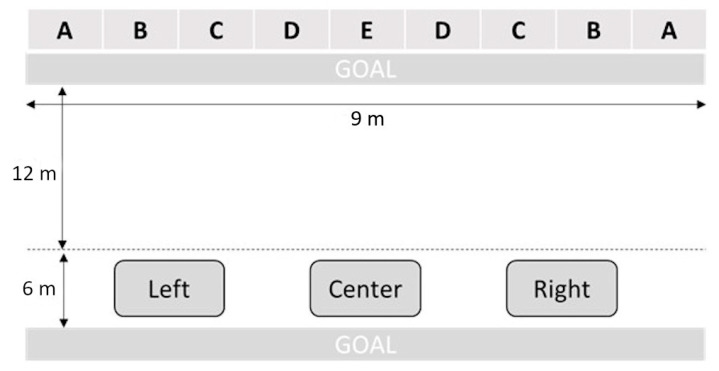
Throwing site (Left, Center, Right) and target areas (A, B, C, D, E) for accuracy test.

The throwing accuracy was evaluated by visual real-time observation, with the observer behind the athlete. It was measured through the analysis of 9 goalball throws: 3 from the right side, 3 from the center, and 3 from the left side of the landing area, with the arm and throwing technique that each player preferred. The goal was divided in nine zones or target areas (1 m per zone), with visual marks in the floor for the researchers (Figure 1). Zones A, C, and D were registered as the places with the highest goal probability, based on previous research [4]. In this way, goals in zones A–C–D were quantified with 2 points, whereas goals in zones B–E were quantified with 1 point. Throws out of goal zones were not quantified (0 points). The global punctuation was the sum of the points in the 9 throwing attempts.

A hand radar gun (ATS II Applied concepts, Dallas, TX, USA) of 50 Hz frequency was used to evaluate the ball velocity during the throws. Its validity measuring ball velocity has been previously reported [21]. The radar gun was placed on a tripod 3 m behind the throwing place and at a 1 m height. Athletes were instructed to shoot in their own time. Velocity values were registered by the radar gun from the beginning of the shooting movement (detected by an increase in speed). Only those throws that ended in a goal were finally considered. The ball velocity of the highest value of the 9 throwing attempts was reported.

### 2.5. Stabilometry

Static balance was assessed using a computerized pressure platform Freemed (Sensor Medica, Rome, Italy). This system is composed of a pressure-sensitive plate with an active surface of 400 × 400 mm and 8 mm thickness. The software used to capture and analyze the data belongs to the platform FreeStep v.1.0.3 (Sensor Medica, Rome, Italy). The reliability of this baropodometric platform has been shown in other studies [22]. Athletes performed the following test: stance for 10 s with a single leg, both left and right, using their blackened goggles for 10 s, performing two attempts/leg [23]. The center of pressure mean velocity (COP-MV) (mm/s) and the surface area (SA) of the stabilogram (mm^2^) were considered in the study.

### 2.6. Core Stability

**Figure 2:**
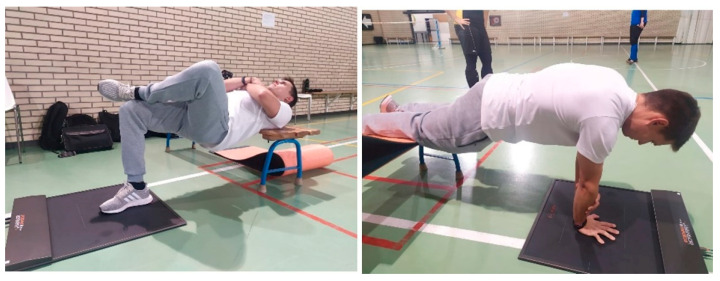
Core stability test positions: single leg hip thrust (**left**) and single arm plank (**right**).

Trunk stability was assessed using the same pressure platform for the static balance test (see ‘Stabilometry’ section). Two static positions were evaluated: single arm plank, with the free arm holding the forearm with the elbow at 90° flexion, and single leg hip thrust (Figure 2), maintaining each position for 10 s. Dominant arm and leg were used for the test [24]. COP-MV and SA were also registered in this assessment.

### 2.7. Acoustic Reaction Test

Acoustic reaction time was assessed using the reaction time reactivity system of Optogait (Microgate SRL, Bolzano, Italy; Software Optogait Version 1.12.21 Microgate SRL, Bolzano, Italy). The optoelectric detection device used in this study consists of two bars (one transmitting and one receiving) of 100 × 8 cm placed parallel to each other and contains 96 light diodes located 3 mm above the floor level. This device has been previously used to assess reaction time [25]. Reaction times were evaluated acoustically using two tests. The first one consisted of an upper limb simple reaction test, where participants tried to lift one hand when they received the acoustic stimulus [26].

**Figure 3:**
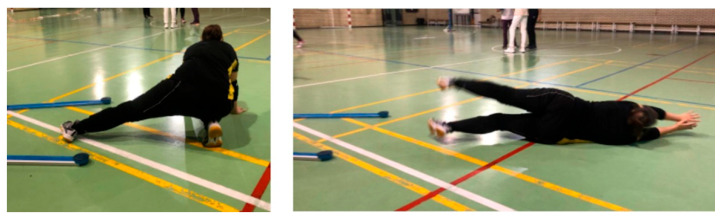
Specific defensive goalball movement from low position with the acoustic stimulus.

The second test consisted of a complex reaction test, where athletes performed a specific defensive goalball movement from low position with the acoustic stimulus (Figure 3). Two training attempts and three experimental tests were carried out, with one minute of rest between tests.

### 2.8. Statistical Analysis

The sampling has been purposeful, so the calculation of the sample size is not appropriate. This calculation proceeds using probabilistic sampling techniques. This situation limits the external validity and the possibility of generalizing the results of the study but does not compromise the internal validity of the results.

Statistical analyses were performed using SPSS version 25.0 for Windows (SPSS Inc., Chicago, IL, USA). Descriptive statistics (median, interquartile amplitude, maximum, and minimum) were calculated for all variables. Normality of datasets was checked with Shapiro–Wilk test.

Three variables (dominant leg COP-MV (*p* = 0.006), dominant leg SA (*p* = 0.036), and single arm plank SA (*p* = 0.046) showed a non-normal distribution. Spearman’s correlation was selected, calculated, and used to determine lineal relationships between all measurements (including descriptive variables). Qualitative criterion for Spearman’s correlation coefficient (±0.001–±0.299 Poor; ±0.300–±0.599 Fair; ±0.600–± 0.799 Moderate; ±0.800–±0.999 Very strong; ±1 Perfect) [24] was applied.

All tests were performed with a level of significance *p* < 0.05. Statistical power (1-β) was analyzed post hoc with G*Power 3.1.9.7 software, to determine the power of the study based on the sample size (*n* = 8) and the calculated effect size (Spearman’s correlation coefficient).

To assess the reliability of acoustic reaction test (simple and complex), intraclass correlation coefficient was applied, establishing < 0.50 poor; 0.5–0.75 moderate; 0.75–0.9 good; and >0.9 excellent reliability [27].

## 3. Results

The main characteristics of the sample are shown in Table 1. Data are expressed as median and interquartile amplitude, showing the maximum and minimum value of each variable. Raw data did not show a significant statistic adjust with normal distribution using the Shapiro–Wilk test. A great heterogeneity can be observed in all anthropometric values.

A positive and statistically significant (*p* < 0.05) correlation was found between ball-throwing velocity and years of experience (Ƿ = 0.714, *p* = 0.047, 1-β = 0.57), height (Ƿ = 0.786, *p* = 0.021, 1-β = 0.72), dominant leg SA (Ƿ = 0.738; *p* = 0.037; 1-β = 0.62), and non-dominant leg COP-MV (Ƿ = 0.714, *p* = 0.017, 1-β = 0.57). Through an a priori power analysis, with an alpha error of 0.05, a beta power of 0.80, and an effect size of 0.714 (minimum statistically significant correlation coefficient found in this study), the number of sufficient subjects required is 12 in total for future studies. All of these correlations were moderate [27], but they show a statistical power less than 0.80.

A good reliability was found in the simple reaction test (ICC = 0.89) and the non-dominant complex reaction time (ICC = 0.75). In the dominant complex reaction time, reliability was poor (ICC = 0.29). Reliability of the gold standard test, as radar gun measures, was not performed.

## 4. Discussion

In this study, the throwing precision and velocity in elite goalball players have been analyzed, studying the relationship with the core stability, balance, anthropometry, and acoustic reaction velocity.

Goalball is a sport with a very small number of licenses in Spain (1098 in season 2020–21), according to the Spanish Federation of Sport for Blinded People [28]. Even a top league team is non-professional, and the volume of training is very low compared with other sports. There is a great heterogeneity of characteristics among players (as shown in Table 1), which makes the research in this field more complicated. As a first step, this study has described the characteristics of a Spanish top league team in terms of anthropometry, balance, acoustic reaction, throwing velocity, and precision.

These variables are well-documented in other sports with similar attacking actions, where a ball is thrown to a goal. In goalball, there are different ways of throwing: traditional shots and rotation shots that are bouncing or flat [4]. In the present study, a positive correlation between throwing velocity and height and experience has been observed. In other sports where players had to perform throwing, experience is also very important. A recent study in beach handball players has found that elite players have a higher throwing velocity [29]. Beach handball allows different way of throwing, including rotation shots, as in goalball, although the technique is quite different. Height is also a key factor, because, from a biomechanical point of view, the lever arm is longer, so the velocity that could be transferred to the ball is higher [30]. Other studies have analyzed the importance of an athlete’s height and throwing velocity in different sports, such as handball [31], water polo [32], baseball [33], and track and field [34,35,36]. As a non-trainable factor, height could be considered in talent detection because of its relationship with ball velocity, as in youth handball players [37]. Throwing velocity in this study is similar to previous research on top league teams, which came from Brazil [2], suggesting that player characteristics are similar in both countries (Spain and Brazil). Further studies should assess how different physical and technical training protocols could improve throwing ability.

Balance is a key factor in throwing ability. As described in the Results section, throwing velocity shows a positive correlation with dominant leg SA and non-dominant leg COP-MV. Perhaps this is the most surprising find in the study, as the authors expected a negative correlation between SA, COP-MV, and throwing velocity. The explanation for this finding is unknown. On the other hand, it is documented that pitchers with lower balance levels (lower vestibular input) had worse precision in throwing [38]. Maybe blinded people, whose balance depends more on the vestibular system, can take advantage of this to improve their throwing velocity. However, this affirmation must be confirmed in future studies. In reference to the system for balance evaluation, other more affordable solutions could be applied such as accelerometers [19].

Precision is the compulsory complement of velocity in goalball throws. In the present study, a novel accuracy test is proposed, with three different zones of throwing and nine different zones of scoring, as previously described (see Methods section). This test results in a number that represents the player’s ability to score, based on the zone with the highest probability of a goal. Precision is an important factor in different sports, so it has been assessed in water polo [39], handball [40], and soccer [41]. The new tool presented here could be very useful to assess the progress of a specific precision training or be interesting for selecting players with more possibilities of scoring during the game.

In addition to throwing effectiveness when attacking, it has been previously described that balance and core stability are related with performance in several sports [42]. In handball players, Caballero et al. [43] found that young players (U12) had worse results in a balance task than older ones (U16 and 18+). They also found that expert players had better performance on a balance task than recreational players. In our study, data showed that years of experience correlates with worse results in COP-MV and SA. Another study with soccer players found that after a balance training period, both visually impaired and not visually impaired players improved their results [44]. Maybe a specific training for goalball players, including balance tasks, would lead to better performance in different game actions such as throwing or defensive moves.

During a goalball game, players change their role from attack to defense continuously. Attack is based on throws, with special importance placed on ball velocity and accuracy. On the other hand, defensive tasks consist of blocking the ball to prevent an opponent’s goal. Players must perform blockage by attending to the sound that the bells inside the ball make, so acoustic reaction time is critical. Until the present day, there has been no research related to this factor in goalball players, but it had been evaluated in other sports such as taekwondo [45], soccer [46], and swimming [47]. The present study analyzed acoustic reaction time and complex acoustic reaction time, showing a better reaction time when the task is simple (0.42 ms vs. 0.69 ms). When the task is more complex, the reaction time is slower [48]. Accordingly, it is important to design a test that is the closest to real-game tasks. Our test proposal for acoustic reaction is based on a specific defense movement from a lower position, which is the most common movement in goalball defense. This test shows a good reliability when applied to the player’s preferred movement, i.e., the non-dominant leg. When the non-dominant leg is inside the measuring area, the movement is more natural, and the player dives to the dominant side. If the test is performed using the same movement to the other side (dominant leg complex time), the reliability is poor, maybe because this movement is not used by players during the game. Spratford et al. [49] found different movement patterns in diving saves to the preferred and non-preferred sides of professional goalkeepers.

The current study presents some limitations. There is a small number of goalball players in our region, so that is why a great variety of level, experience, age, etc., is observed in the variables analyzed. According to the data presented, 12 subjects should be recruited in future studies. A homogenous sample could provide more consistent information. The familiarization absence in some tasks could be considered as another limitation, but the lack of experience was necessary to avoid specific learning effects.

## 5. Conclusions

To conclude, to the best of our knowledge, this is the first test proposal to assess throwing precision and complex acoustic reaction in goalball players. These tests focus on the performance factors of goalball, so they could be used to assess the level of performance in future studies, both descriptive of other playing levels and pre–post studies about training interventions. It has also been shown that, in this goalball team, height and years of experience are the most important factors related to throwing the ball with the highest ball velocity, being independent of core stability and balance. According to data from other sports, maybe specific core and balance training could improve both the precision and velocity of throwing in goalball. The test proposed in this study could be a useful evaluation of the tool for the performance parameters that determine the throwing ability and defense for goalball trainers.

## Figures and Tables

**Table 1:** Physical characteristics and performance test results (*n* = 8).

Variable	Md	IA	Max	Min
Age (years)	31	19	45	20
Weight (kg)	76.0	45.0	110.0	47.0
Height (cm)	171	26	196	159
BMI (kg/m^2^)	25.4	8.5	30.1	18.5
Experience (years)	9	10	18	1
Dominant leg COP-MV (mm/s)	48	133.7	189.4	20.7
Dominant leg SA (mm^2^)	2243.2	14,582.2	21,926.1	679.8
Non-dominant leg COP-MV (mm/s)	70.6	51.6	124.5	26.6
Non-dominant leg SA (mm^2^)	2606.6	3887.3	7466.6	876.4
Single arm plank COP-MV (mm/s)	3.3	3.1	7.71	2.6
Single arm plank SA (mm^2^)	6.7	21.3	30.7	2.6
Single leg hip thrust COP MV (mm/s)	8.8	7.3	15.3	2.4
Single leg hip thrust SA (mm^2^)	95.7	234.1	365.2	12.7
Ball velocity (km/h)	50.7	22.6	68	33.4
Throwing accuracy (unitless)	13	2	16	9
Simple ART (ms)	0.4	0.22	0.6	0.3
Dominant leg complex ART (ms)	0.7	0.17	0.9	0.6
Non-dominant leg complex ART (ms)	0.6	0.14	0.8	0.6

BMI = body mass index; Md = median; IA = interquartile amplitude; COP-MV = center of pressure mean velocity; SA = surface area; ART = acoustic reaction time.

## Data Availability

Not applicable.
